# Alternative Splicing Dynamics of the Hypothalamus–Pituitary–Ovary Axis During Pubertal Transition in Gilts

**DOI:** 10.3389/fgene.2021.592669

**Published:** 2021-04-30

**Authors:** Xiangchun Pan, Qingnan Li, Danxia Chen, Wentao Gong, Nian Li, Yao Jiang, Hao Zhang, Yaosheng Chen, Xiaolong Yuan

**Affiliations:** ^1^Guangdong Provincial Key Lab of Agro-Animal Genomics and Molecular Breeding, National Engineering Research Center for Breeding Swine Industry, College of Animal Science, South China Agricultural University, Guangzhou, China; ^2^State Key Laboratory of Biocontrol, Guangzhou Higher Education Mega Center, School of Life Sciences, Sun Yat-sen University, Guangzhou, China; ^3^Guangdong Provincial Key Laboratory of Laboratory Animals, Guangdong Laboratory Animals Monitoring Institute, Guangzhou, China

**Keywords:** alternative splicing, differentially spliced genes, hypothalamus-pituitary-ovary axis, gilts, puberty

## Abstract

The timing of puberty in mammals marks the point at which reproduction becomes possible. Abnormalities in the timing of puberty may exert a series of negative effects on subsequent health outcomes. Alternative splicing (AS) has not only emerged as a significant factor in the transcription of genes but it is also reported to play a role in the timing of puberty. However, to date, the changes and dynamics of AS during the onset of puberty is extremely seldom explored. In the present study, we used gilts as a research model to investigated the dynamics of AS and differentially expressed AS (DEAS) events within the hypothalamus–pituitary–ovary (HPO) axis across pre-, in-, and post-puberty. We detected 3,390, 6,098, and 9,085 DEAS events in the hypothalamus, pituitary, and ovary when compared across pre-, in-, and post-pubertal stages, respectively. Within the entire HPO axis, we also identified 22,889, 22,857, and 21,055 DEAS events in the pre-, in-, and post-pubertal stages, respectively. Further analysis revealed that the differentially spliced genes (DSGs) associated with staged DEAS events were likely to be enriched in the oxytocin signaling pathway, thyroid hormone signaling pathway, GnRH signaling pathway, and oocyte meiosis signaling pathway. The DSGs associated with DEAS events across the entire HPO axis were enriched in endocytosis signaling pathway, the MAPK signaling pathway, and the Rap1 signaling pathway. Moreover. the ASs of *TAC1*, *TACR3*, *CYP19A1*, *ESR1*, *ESRRA*, and *FSHR* were likely to regulate the functions of the certain HPO tissues during the onset of puberty. Collectively, the AS dynamics and DEAS events were comprehensively profiled in hypothalamus, pituitary, and ovary across the pre-, in-, and post-pubertal stages in pigs. These findings may enhance our knowledge of how puberty is regulated by AS and shed new light on the molecular mechanisms underlying the timing of puberty in mammals.

## Introduction

The timing of puberty in females relates to the first ovulation and the attainment of reproductive capability (Euling et al., 2008). Abnormalities in the timing of puberty exert a series of different diseases (Cesario and Hughes, 2007), including psychosocial disorders (Hankin et al., 2010), asthma (Al-Sahab et al., 2011), tumors of the reproductive system (Bodicoat et al., 2020), and hypogonadism (Abreu and Kaiser, 2016). Previous studies have demonstrated that the hypothalamic–pituitary–ovary (HPO) axis is responsible for controlling the timing of puberty in females (Zhu et al., 2019). During the onset of puberty, the hypothalamus releases gonadotropin-releasing hormone (GnRH) to the pituitary gland. This process promotes the release of follicle-stimulating hormone (FSH) and luteinizing hormone (LH) into the circulating system (Plant, 2015a). Subsequently, FSH and LH act to accelerate ovarian folliculogenesis and regulate the ovulation of mature follicles (Plant, 2015b; Avendaño et al., 2017; Zhu et al., 2019). Previous researchers have found that GnRH deficiency can cause delayed puberty (Balasubramanian et al., 2010). Furthermore, girls with abnormal levels of LH and FSH are associated to reduced ovarian weight and pubertal failure (Stagi et al., 2016). In rats, the disruption of follicular dynamics results in a delay in the onset of puberty (Lilienthal et al., 2006). These observations indicate that abnormalities in the HPO may lead to pubertal disorders, which in turn has a negative effect on health outcomes.

Alternative splicing (AS) is a post-transcriptional processing mechanism that assembles multiple transcripts with different functions from a single gene under the action of the spliceosome (Lee and Rio, 2015). AS determines whether exons are retained or skipped, excluded or included, and shortened or extended to form mature mRNA (Carazo et al., 2019). There are evidence reported that AS plays a vital role during the development of the hypothalamus (Hasin-Brumshtein et al., 2016), pituitary (Vazquez-Borrego et al., 2019), ovary (Wang et al., 2015), and estrus (Tang et al., 2018). For example, a previous study has shown that piRNAs regulate the pre-mRNA splicing of transcripts and may result in the production of a non-transposase-encoding mature mRNA isoform in germ cells (Teixeira et al., 2017). Also, other researchers have identified two alternative transcripts of the *Lin28B* gene (*Lin28BS*, encoding 247 amino acids (aa); and *Lin28BL*, encoding 261 aa) that regulate the timing of puberty in mammals (Cao et al., 2013). Similarly, another study has discovered that two *Oct-2* transcripts (*Oct-2a* and *Oct-2c*) act as control mechanisms in the hypothalamus and regulate female puberty; these transcripts can act rapidly and alternatively, thus exerting control on the onset of puberty in the female mammal (Ojeda et al., 1999). Moreover, two isomers of the *KISS1R* gene (*Ss kiss1r_v1* and Ss *kiss1r_v2*) have been shown to act as the gatekeeper for the onset of puberty, which also exhibit different functions during puberty (Mechaly et al., 2009). Collectively, these findings indicate that the different splicing types caused by AS may have an impact influence on the onset of puberty. However, little is known about the AS events that regulate the onset of puberty.

The rapid development of next-generation sequencing (NGS) technology has led to an increasing number of research studies on sequence-based AS. A previous study used RNA-sequencing (RNA-seq) to detect AS by accurately measuring the percent spliced-in (PSI) score (Hardwick et al., 2019). Another study also used RNA-seq to find that when a mutation occurred at a splice site, the proteins became non-functional (Mayerle et al., 2019). Furthermore, other researchers translated the sequences of new splice junctions, derived from RNA-seq into analogous polypeptide sequences, and created a database that can be used to discover new splice junction peptides that arise from AS (Sheynkman et al., 2013). In another study, researchers used long read sequencing to reveal the splicing status of introns in yeast (Oesterreich et al., 2016). Subsequently, other researchers developed an AS platform for regulating exons that undergo mutually exclusive exons (MXE) AS, ultimately identifying a functionally diverse range of RNA and protein isotypes (Mathur et al., 2019). In addition, the action of the splicing factors (SFs) as “scissors” can exert significant influence on AS. When the components of the SFs undergo alterations, it is likely to affect the occurrence of AS (Zhang et al., 2008). These results indicate that with the development of NGSs, the research on AS deserves more attention.

In the present study, we used gilts as a model and collected HPO tissues from pre-, in-, and post-pubertal animals. Then, we built strand-specific RNA libraries from the HPO tissues and investigated differentially expressed AS (DEAS) events and AS dynamics within the pubertal hypothalamus, pituitary, and ovary. Furthermore, we analyzed the comprehensive changes and dynamics of AS across pre-, in, and post-pubertal stages, as well as along the HPO axis. Finally, we predicted the interactions between several SFs and specifically spliced genes events might associated with puberty. This study may provide new insights into the mechanisms underlying the timing of puberty in female mammals, particularly with regards to AS.

## Materials and Methods

### Ethics Statement

Animal care and experiments were conducted in accordance with the regulations for the administration of affairs concerning experimental animals (Ministry of Science and Technology, China; revised in June 2004) and were approved by the Animal Care and Use Committee of the South China Agricultural University, Guangzhou, China (permit number SCAU#2013-10).

### Preparation of Animals and Samples

Pre-, in-, and post-pubertal stages were chosen during the onset of puberty; these different stages were identified by measured visually the reddening, swelling of the vulva, and by analyzing standing reflex with the back-pressure test and boar contact (Patterson et al., 2002). Nine Landrace × Yorkshire crossbred gilts with good body condition were used in this study. All gilts were raised in a clean, dry, and well-lit room with equal conditions and maintained on a standard normal diet (isoenergetic corn–barley and soybean meal diet) and water *ad libitum*. Three gilts without pubertal signs (no reddening, no swelling of the vulva, no standing reflex) were selected as the pre-pubertal gilts (age = 162 ± 3 days; weight = 81.38 ± 2.40 kg), and three gilts exhibiting the first pubertal signs (reddening, swelling of the vulva, standing reflex) were selected as the in-pubertal gilts (age = 212 ± 14 days; weight = 110 ± 2 kg). Gilts have regular estrus cycles, typically 18–23 days (Christenson and Ford, 1979). We used 14 days after in-puberty as the post-puberty, and this stage was diestrus in entire estrus cycles. Finally, three post-pubertal gilts were selected (age = 216 ± 17 days; weight = 122.82 ± 9.11 kg). All gilts were euthanized with an intravenous overdose of barbiturates (≥90 mg/kg). Subsequently, the hypothalamus, entire pituitary and ovary (left and right) were immediately removed. Immediately after decapitation, to dissect the hypothalamus regions, the cerebellum was removed to expose the junction of the brainstem and the cerebrum, so that the hypothalamus is visible, and the hypothalamus is cut along the root as well as the entire pituitary is taken from the pituitary fossa. Moreover, left and right ovaries were taken out. Meanwhile, the biological characteristics of these three stages were reconfirmed by the morphology of ovary (Supplementary Figure 1). All tissues were snap-frozen in liquid nitrogen and stored at −80°C to await subsequent sequencing.

### RNA Sequencing, Quality Control, and Transcriptome Assembly

Tissues of pre-, in-, and post-pubertal hypothalamus, pituitary, and ovary were homogenized separately in liquid nitrogen. Total RNAs were extracted from tissue extracts using Trizol (Invitrogen, Carlsbad, CA, United States). Quality control tests were carried out on the RNA using an Agilent Bioanalyzer 2100 system (Agilent, Palo Alto, CA, United States). Then, we used an Epicenter Ribo-zero rRNA Removal Kit (Epicenter, Madison, WI, United States) to remove rRNAs; total RNAs were subsequently quantified. After total RNA extraction and treatment with DNase I, we next used magnetic beads and oligo (dT) to extract mRNAs. Fragmentation buffer was then added to the purified mRNAs to cause fragmentation. Next, we used random hexamer primers and the mRNA fragments as templates to synthesize cDNAs. DNA fragments were then adenylated at the 3’-ends and then ligated to adapters. We then used cDNA fragments (100–200 bp in length) and PCR to generate cDNA libraries. A total of 5 μg of cDNA per sample was sequenced using the HiSeq 3000 Sequencer; this system was operated as recommended by the manufacturer (Illumina, San Diego, CA, United States) and 150 base paired-end reads were generated. The quality control of the raw and trimmed reads was performed using FastQC and Cutadapt software. FastQC software checked the quality of directional paired-end reads in the raw data (Ward et al., 2020). Cutadapt software discarded the low-quality reads (unknown bases >10%; low-mass bases > 50%) and the adapter contamination to generate the high-quality reads termed as clean data thereafter (Chen et al., 2014). Following quality control, the clean reads were mapped to *Sus scrofa* 11.1 by HISAT2 software (Kim et al., 2015).

### Identification of DEAS Events

The PSI value is an important index with which to identify AS events; significant pairwise differences can be identified using PSI values (| delta-PSI [ΔPSI]| ≥ 10%; FDR ≤ 5%) (Sandberg et al., 2008). The PSI metric represents the ratio of normalized read counts and indicates exon inclusion as a fraction of the total normalized reads for both exon inclusion and exon exclusion (Pervouchine et al., 2013). In addition, CASH software that can significantly improve the detection of DEAS events between samples^1^ (Wu W. et al., 2018). Moreover, CASH can directly identify different AS events by considering the value of ΔPSI as well as providing exon inclusion and exon exclusion reads (Wu W. et al., 2018). Therefore, in the present study, we primarily used CASH software to identify staged DEAS events across pre-, in-, and post-pubertal tissues, and to identify DEAS events across different tissues in the HPO axis. Then, we calculated the PSI using normalized reads while considering inclusion and exclusion data (FDR < 0.05). We used two specific modules in CASH software: SpliceCons and SpliceDiff. The first module detects eight different types of AS events that are classified according to splicing at different sites, including alternative 3’ splice site (A3SS), alternative 5′ splice site (A5SS), mutually exclusive exons (MXE), cassette exon (cassette, SE), multi-cassette exons (Cassette multi), intron retention (IR), alternative start exon (AltStart), and alternative end exon (AltEnd); these are depicted in Supplementary Figure 1. Then, we used the second module, SpliceDiff, to calculate the ΔPSI between each sample and thus identify DEAS events.

### Analysis of DEAS and DSG Data

DEAS were identified using ΔPSI (FDR < 5%), and differentially spliced genes (DSGs) were defined as the parental genes from DEAS events. We also used R (version 3.5.1) to perform statistical analysis. Specifically, we used the functions of setdiff and intersect to analyze the number of genes undergoing AS. Next, we used the Venn Diagram package (Chen and Boutros, 2011) and the ggplot2 package (Villanueva and Chen, 2019) to visualize data. Upset plots were created using TBTools version 0.66836 software^2^. Stage-specific AS events were defined as those that only occurred in one pubertal stage with the screening condition of | △PSI| = 1. Similarly, tissue-specific AS events were defined as those that only occurred in one tissue with the screening condition of | △PSI| = 1 and FDR < 0.05. Finally, statistical differences among multiple groups were identified by ANOVA with Tukey’s multiple comparison test.

### KEGG Pathway Enrichment and Splicing Factor Analysis

Next, DSGs were used for Gene Ontology (GO) analysis and Kyoto Encyclopedia of Genes and Genomes (KEGG) pathway enrichment analysis using the FDR < 0.05 as a cutoff criterion. Co-existing DSGs were identified with KOBAS 3.0 online software. Furthermore, a list of splicing factors (SFs) was extracted from the SpliceAid-F database^3^; this database supplies the gene interactors of SFs (Giulietti et al., 2013). Finally, we used Cytoscape software (version 3.4.0) to generate a network of protein interactions between SFs and genes that are known to be associated with puberty.

### RT-PCR Analysis

Finally, we used RT-PCR to validate the reliability of the data of RNA-seq. Upstream and downstream primers were designed from the upper exon and lower exon of the target splicing region using NCBI primer designing tool. PCR amplification was performed using cDNA as a template. The PCR standard procedure was denaturation 94°C (3 min), 35 cycles at 94°C (30 s), 60°C (30 s), and 72°C (30 s), subsequently extending it for 10 min at 72°C. The PCR products were detected via running 2.5% agarose electrophoresis. All reactions were repeated three times.

## Results

### Identification of AS Events in HPO Tissues During Pubertal Transition in Gilts

In total, we generated 27 RNA-seq libraries consisting of nine hypothalamic tissues (611 million clean reads), nine pituitary tissues (673 billion clean reads), and nine ovarian tissues (597 million clean reads) obtained from gilts undergoing pubertal transition. These clean reads were subsequently mapped to the *Sus scrofa* reference genome by using HISAT2 with an average alignment rate of >90% (Supplementary Table 1).

**FIGURE 1:**
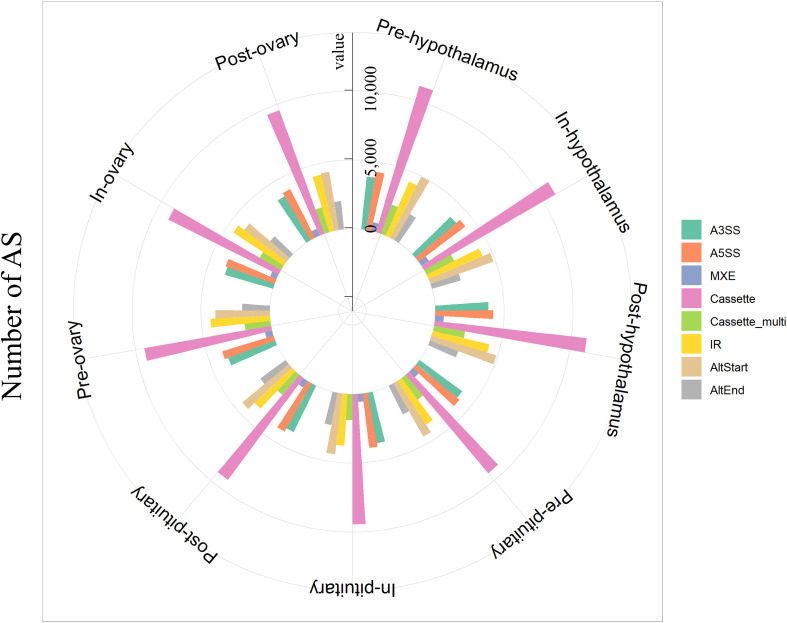
Overview of alternative splicing (AS) events in the pubertal hypothalamus–pituitary–ovary (HPO) axis. The number of AS events in the hypothalamus, pituitary, and ovary of gilts during pre-, in-, and post-pubertal stages.

In the present study, eight types of AS events were investigated in total, including A3SS, A5SS, MXE, Cassette, Cassette multi, IR, AltStart, and AltEnd events (Supplementary Figure 2). A total of 278,721 AS events were detected in pubertal HPO tissues across pre-, in-, and post-pubertal tissues (FDR < 0.05). In total, 33,535, 33,254, and 33,405 AS events were detected in the hypothalamus during pre-, in-, and post-pubertal stages, respectively (Figure 1 and Supplementary Tables 2, 3); 30,251, 30,135, and 29,767 AS events were identified in the pituitary during pre-, in-, and post-pubertal stages, respectively (Figure 1 and Supplementary Tables 2, 4); and 29,567, 28,684, and 30,123 AS events were detected in ovarian tissue during pre-, in-, and post-pubertal stages, respectively (Figure 1 and Supplementary Tables 2, 5). The most common AS event was Cassette events, while the least common was MXE events in all stages of HPO axis (Supplementary Table 6 and Supplementary Figure 3A, Tukey’s test). Collectively, these data indicate that we identified eight AS events during different pubertal stages in HPO tissues.

### Differentially Expressed AS Events During Different Stages of Puberty Transition

**FIGURE 2:**
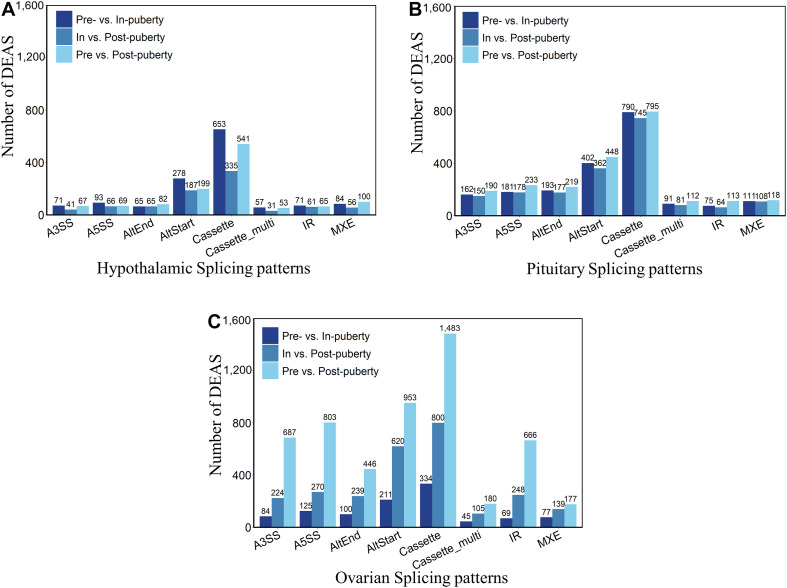
Staged differentially expressed alternative splicing (DEAS) events in the hypothalamus–pituitary–ovary (HPO) axis. Pairwise comparisons detected DEAS events in the hypothalamus **(A)**, pituitary **(B)**, and ovary **(C)** of pre-, in-, and post-pubertal tissues.

To explore the DEAS events in HPO tissues during different stages of pubertal transition, we carried out pairwise comparisons across the pre-, in-, and post-pubertal groups in three tissues from the HPO axis. The distribution of DEAS events is shown in Figure 2. When comparing pre- vs. in-puberty, in- vs. post-puberty, and pre- vs. post-pubertal groups, we detected 3,390 DEAS events in the hypothalamus (Figure 2A), 6,098 DEAS events in the pituitary (Figure 2B), and 9,085 DEAS events in the ovaries (Figure 2C). The most frequent DEAS in the hypothalamus and pituitary were Cassette events (Supplementary Table 7 and Supplementary Figures 3B,C, Tukey’s test). Pairwise comparisons of pre- and post-pubertal groups showed the highest frequency of DEAS events (Supplementary Figure 3D, Tukey’s test). In total, there were 18,573 DEAS involved in the pre-, in-, and post-pubertal HPO axis. Further analysis showed that the prevalence of staged DEAS was different when compared across the three tissues, thus indicating that different AS events might play various roles in the pubertal HPO axis.

### DSGs in Different Stages of Puberty and KEGG Analysis

**FIGURE 3:**
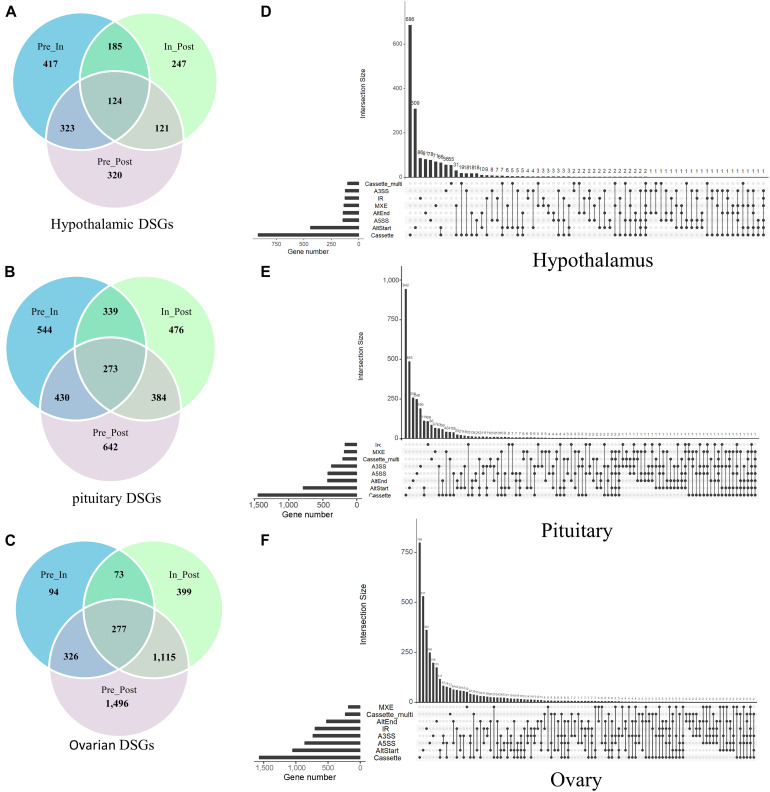
Characterization of the parental genes for staged differentially expressed alternative splicing (DEAS) events. The number of parental genes of the DEAS detected in the hypothalamus **(A)**, pituitary **(B)**, and ovary **(C)**. Upset plot of different types of DEAS events, and their parent genes, in the hypothalamus **(D)**, pituitary **(E)**, and ovary **(F)**. The abscissa is the number of genes that have occurred corresponding to AS, the ordinate represents the number of genes that occurred multiple AS. One gene may have up to six types of alternative splicing.

In total, we identified that 1,737 DSGs in the hypothalamus that were the parental genes of DEAS (Figure 3A), 3,088 DSGs in the pituitary (Figure 3B), and 3,780 DSGs in the ovaries (Figure 3C). It is noteworthy that several AS events might occur in the same gene. Specifically, an upset plot of multiple interactive sets demonstrated that 14.29% (Figure 3D), 22.72% (Figure 3E), and 33.54% (Figure 3F) of DSGs featured two or more DEAS events in the three tissues. In summary, these results showed that DEAS events might provide the possibility for improving diversity within the transcriptome.

**FIGURE 4:**
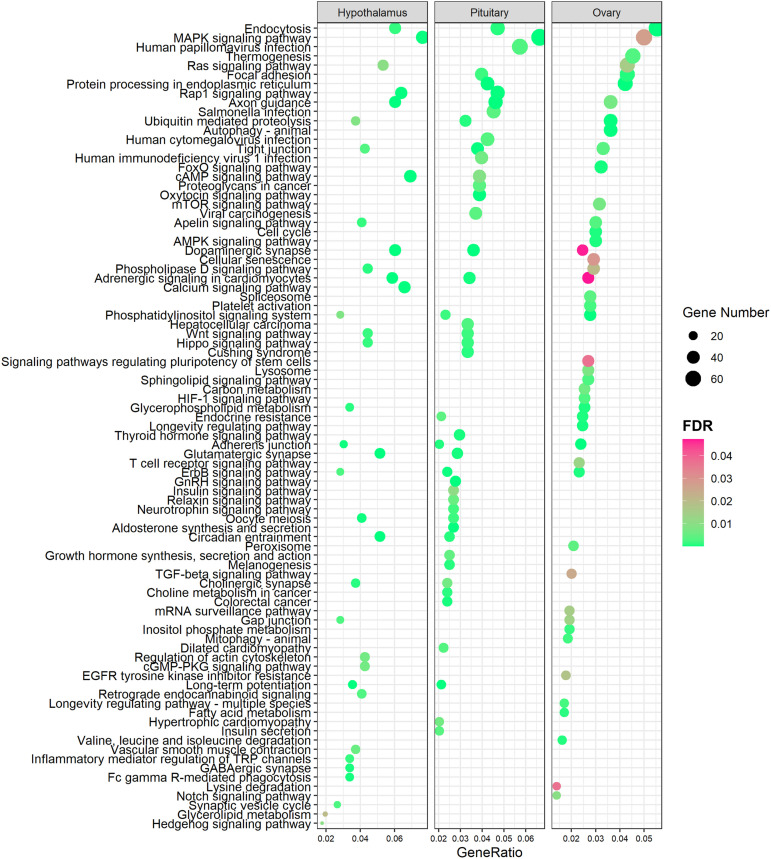
KEGG analysis of differentially spliced genes in three tissues from gilts (FDR < 0.05).

To determine the biological functions of these staged DEAS events within the HPO axis, we performed KEGG enrichment analysis for the DSGs of DEAS events in the HPO axis during different stages of puberty. KEGG enrichment analysis showed that DSGs in the three tissues showed enrichment in different pathways (Supplementary Tables 8–10). DEGs were associated with the oxytocin signaling pathway, GnRH signaling pathway, insulin signaling pathway, thyroid hormone signaling pathway, and neurotrophin signaling pathway in the pituitary (Figure 4). Moreover, the oocyte meioses were enriched both in the hypothalamus and the pituitary. We also found that the tight junction was enriched in all three tissues (Figure 4).

### Differentially Expressed AS Events in Different HPO Tissues During Pubertal Transition

**FIGURE 5:**
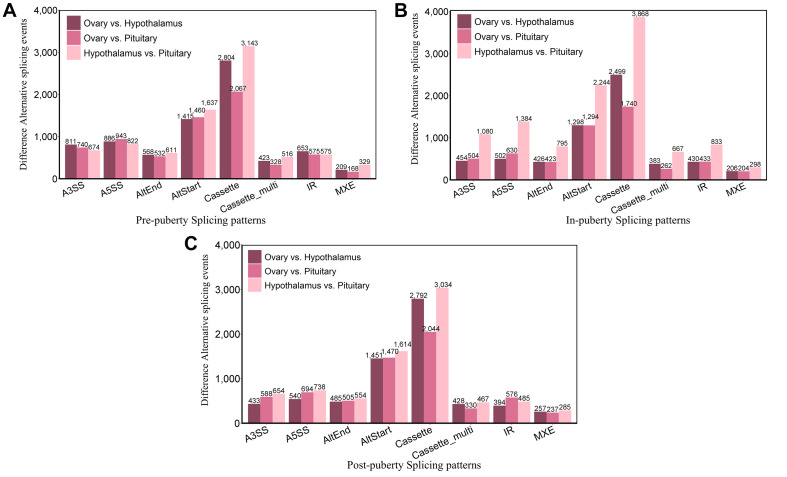
Tissular differentially expressed alternative splicing (DEAS) events during pubertal transition. Pairwise comparison–detected DEAS events were detected pre-puberty **(A)**, in-puberty **(B)**, and post-puberty **(C)** in the hypothalamus, pituitary, and ovary.

Next, we investigated DEAS events in different HPO tissues during pubertal transition in gilts. AS events were compared between the three different tissues by pairwise comparisons during the three stages of puberty. When comparing the hypothalamus *vs.* pituitary, pituitary *vs.* ovary, and hypothalamus *vs.* ovary, we identified a total of 22,889, 22,857, and 21,055 DEAS events in the pre-, in-, and post-pubertal stages, respectively (Figures 5A–C). The most frequent DEAS events during pre- and post-puberty were Cassette events; however, this event was not significant in the in-pubertal stage (Supplementary Table 11 and Supplementary Figures 2E,F, Tukey’s test). In total, we detected 66,801 DEAS events in different HPO tissues. The differences between tissues with regards to AS patterns indicate that AS might play a different role in different HPO tissues during pubertal transition.

### DSGs in Different HPO Tissues and KEGG Analysis

**FIGURE 6:**
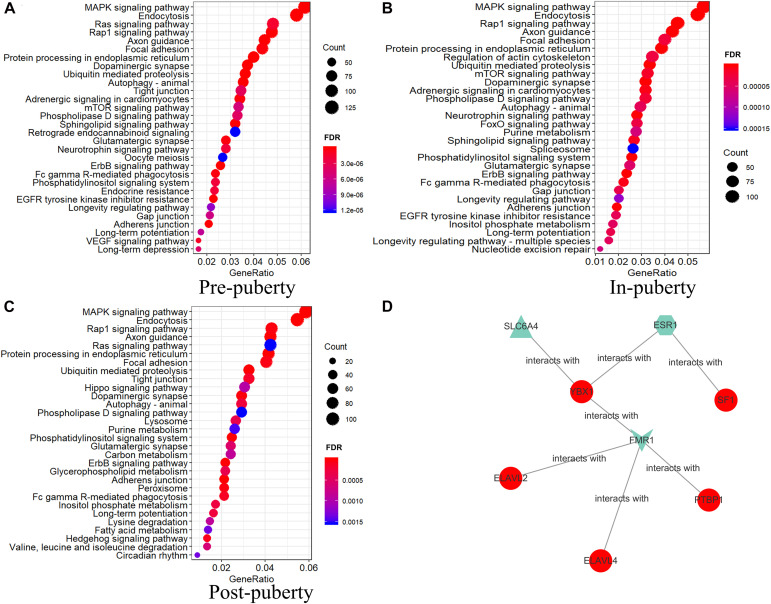
KEGG analysis of tissular differentially spliced genes and a network of splicing factors. KEGG analysis of DEAS in pre-puberty **(A)**, in-puberty **(B)**, and post-puberty **(C)** (FDR < 0.05). **(D)** Network of splicing factors (SFs) and pubertal genes of specific AS. Blue nodes represent stage-specific; purple nodes represent tissue-specific; and red nodes represent both stage- and tissue-specific AS.

To characterize the biological functions of DEAS in different HPO tissues, we performed KEGG enrichment analysis for the DSGs of DEAS events in different HPO tissues across the three stages of puberty. We identified the top 30 significantly enriched pathways (Figure 6). Analysis of the three stages of puberty revealed that the DSGs were significantly enriched in endocytosis, the MAPK signaling pathway, the Rap1 signaling pathway, and axon guidance, in three stages (Figure 6). In particular, several tissular DSGs were enriched in the oocyte meiosis pathway during the pre-pubertal stage (Figure 6A and Supplementary Table 12). Some tissular DSGs were enriched in the spliceosome pathway during the in-puberty stage (Figure 6B and Supplementary Table 13). Finally, some tissular DSGs were enriched in the lysosome and peroxisome pathways during the post-pubertal phase (Figure 6C and Supplementary Table 14).

### DEAS Events in Pubertal Genes

Next, we selected 36 pubertal genes and investigated how these genes underwent DEAS events. To do this, we screened literature and databases by hand. During the pubertal transition in pigs, as shown in Supplementary Table 15, *AVP*, *BDNF*, *MSMB*, and *TAC1* genes respectively produce the A5SS, AltStart, and MXE of AS exclusively in the hypothalamus; *FMR1*, *NCOA1*, *NOS1*, *PGR*, *TACR3*, and *TGFHR1* genes harbor the Cassette, AltStart, and A3SS of AS specially in the pituitary; and *ADM*, *CYP19A1*, *DICER1*, *ENPP2*, *ESRRA*, *GDF9*, *PPARG*, *RIPK2*, and *STAT5B* genes mainly have the IR, AltEnd, AltStart, A3SS, and Cassette of AS in the ovary (Supplementary Table 15). Besides, we found that 16 genes underwent only one AS event: *ADM*, *APOE*, *APP*, *AVP*, *COMT*, *BDNF*, *FMR1*, *FSHR*, *MSMB*, *NOS1*, *NR5A1*, *PPARG*, *PTPN11*, *SLC6A4*, *STAT5A*, and *TACR3*. These 16 genes were subjected to IR, Cassette, AltStart, A5SS, AltEnd, and MXE DEAS events, respectively. However, other genes exhibited complex DEAS events, such as *ESR1* mediated by AltEnd, AltStart, A5SS, and Cassette events. In addition, 20 genes underwent DEAS only in certain tissues, including *ADM*, *AVP*, *BDNF*, *COMT*, *CYP19A1*, *DICER1*, *ENPP2*, *ESR2*, *ESRRA*, *FMR1*, *GDF9*, *MSMB*, *NCOA1*, *NOS1*, *PGR*, *PPARG*, *PIPK2*, *STAT5B*, *TACR3*, and *TGFBR1.* We also found that *APOE*, *COMR*, *ESRRA*, *FSHR*, *NOS1*, *MSMB*, *SLC6A4*, *PGR*, and *NR2C2* only exhibited DEAS events in specific stages. These results demonstrated that diverse patterns of AS events occurred in a range of genes associated with puberty.

### Specific AS Events and Splicing Factor Analysis

To investigate stage-specific and tissue-specific AS events in pubertal gilts, we screened our libraries for specific AS events (| △PSI| = 1, FDR < 0.05). We found that several stage-specific AS events were uniquely expressed in pre-, in-, and post-pubertal stages, of which specific MXE events repeatedly occurred (Supplementary Figure 4A). Similarly, some AS events were uniquely expressed in the three tissues, of which AltStart events repeatedly occurred (Supplementary Figure 4B). Notably, we found that the same gene could experience specific AS events at different sites (Supplementary Tables 16, 17).

To explore the relationship between SFs and specific AS, we went to screen the parental genes of specific AS that interacted with SFs by databases (Supplementary Table 18). We found that multiple SFs interact with specific splicing genes. For instance, the AS of *ACTB* in ovary is specific AltEnd, and SFs Sam68, hnRNP D, YB-1, etc. protein were predicted to interact with *ACTB* (Figure 6D and Supplementary Table 18). Moreover, the AS of *RPS3* is exclusive A3SS, and SFs YB-1, hnRNP U, SLM-1 etc. protein were predicted to interact with *RPS3* (Figure 6D and Supplementary Table 18). According to this analysis, several SFs were predicted to interact with specific spliced genes in specific stage or specific tissue. Future research should focus on investigating interactions between SFs and specific AS events.

### RT-PCR Validation of DEAS Genes

**FIGURE 7:**
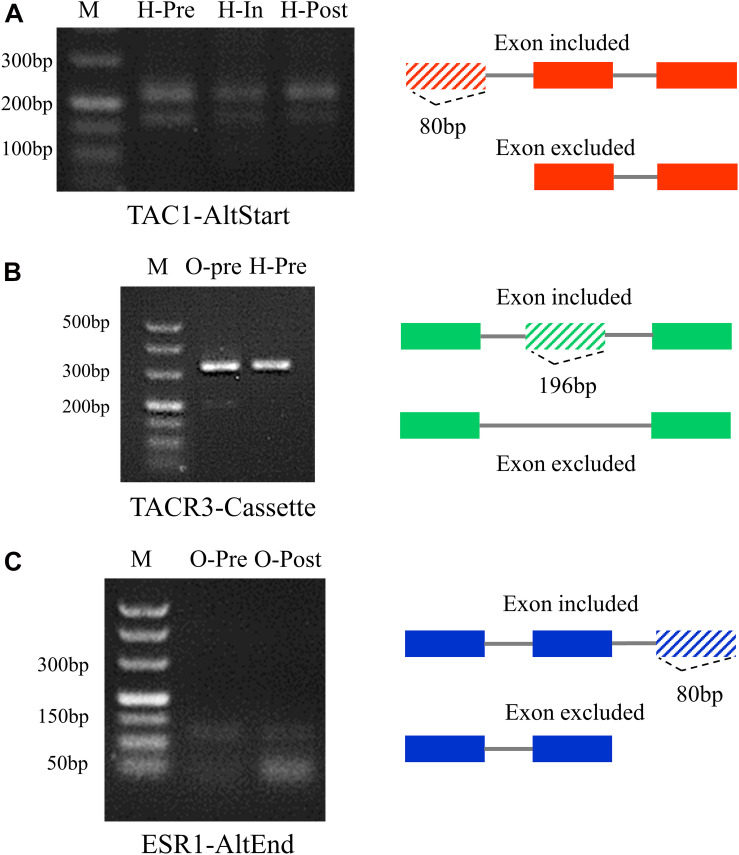
RT-PCR of alternative splicing (DEAS) events for validation. **(A)** DEAS of *TAC1* in pre-, in-, and post-pubertal hypothalamus. **(B)** DEAS of *TACR3* in pre-pubertal ovary and hypothalamus. **(C)** DEAS of *ESR1* in pre- and post-pubertal ovary.

To validate the reliability of the high-throughput RNA sequencing data, three puberty-related DEAS genes were randomly selected for validation experiments. RT-PCR was used to measure the expression of different transcripts that occurred in DEAS events. The primer from the upper and lower exon of the alternative exon was designed (Supplementary Table 19). Among them, *TAC1* occurred different AltStart events of AS in pre-, in-, and post-puberty in hypothalamus, and the phenomenon of the first exon being jumped was most obvious in the pre-puberty (Figure 7A). *TACR3* occurred in different Cassette events of AS in pre-pubertal hypothalamus and ovary, and the phenomenon of the exon being jumped was most obvious in the pre-pubertal ovary (Figure 7B). *ESR1* occurred in different AltEnd events of AS in pre-pubertal ovary and post-pubertal ovary, and the phenomenon of the last exon being jumped was most obvious in the post-pubertal ovary (Figure 7C). The RT-PCR results showed that most of the AS events were in line with our prediction, confirming the reliability of sequencing. Further study should be focus on puberty-related DEAS.

## Discussion

Puberty marks the achievement of sexual maturity and fertility and is mainly driven by the hypothalamic–pituitary–gonadal axis (Blakemore et al., 2010). Moreover, puberty is a very important biological process, irrespective of whether we consider reproduction, development, or disease (Nonneman et al., 2016; Vijayakumar et al., 2018). An increasing body of evidence now indicates that AS may be involved in regulating the function of HPO tissues and the onset of puberty in mammals (Ojeda et al., 1999; Mechaly et al., 2009; Cao et al., 2013). Previous studies have demonstrated that AS plays a crucial role in mammalian development, particularly AS events involving the *ICE* gene (Wang et al., 1998), the *Bcl-2* gene family, the *Ced-4* gene, and the family of *caspase* genes during the process of cellular apoptosis (Jiang and Wu, 1999). However, the AS profiles of tissues in the HPO axis have not been identified during pubertal transition in mammals. In this study, we systematically profiled AS events and analyzed DEAS events in pubertal HPO tissues from gilts. First, we found that the Cassette event was the most common form of AS in pubertal HPO tissues, which is in line with previous analysis of the human transcriptome showing that Cassette events were the most common form of AS (Sultan et al., 2008). Indeed, it has been reported that Cassette is the most common AS event due to the loss of functional domain/sites or the transfer of open reading framework, and the disruption of Cassette is one of the causes of mammalian disease (Kim et al., 2020). Next, we investigated AS events in different tissues of the HPO axis, and during different stages of puberty. We found that tissular DEAS events were much more than staged DEAS events, indicating that the degree of AS is greater between tissues. It is possible, therefore, that the underlying reason that the three tissues of HPO axis play different roles during puberty can be revealed by AS.

AS events exhibit spatiotemporal specificity, which means that AS events vary across different tissues or different stages of development (Chakradhar, 2018). Our DEAS analysis showed that the Cassette event was the most frequent DEAS event in the hypothalamus and the pituitary, but not in the ovary, indicating that AS events might play different regulatory roles within the pubertal HPO axis. We found that the staged DEAS events were likely to occur on genes involving the signaling pathways that have all been reported to regulate the timing of puberty (Parent et al., 2008; Sun et al., 2009; Singh et al., 2015; Aliberti et al., 2019; Gioacchini et al., 2019). For example, previous studies have shown that tight junctions form in cells to create a major component of the blood–testis barrier during puberty in males (McCabe et al., 2012), and the blood–brain barrier also plays a crucial role in the microenvironment required to maintain neuronal function at puberty (Seker et al., 2016). Furthermore, growing ovaries that ingest ovoproteins from the blood stream were mediated by receptor-dependent endocytosis (Mizuta et al., 2013). These observations indicate that genes may be involved in regulating onset of puberty in certain different splicing patterns during mammalian puberty. According to our analysis of DEAS events within the entire HPO axis, one interesting finding is that the transcription of HPO tissues might undergo dramatic changes during the in-puberty stage. Furthermore, we found that DEAS events at the tissue level were more likely to occur on genes related to signaling pathways that have all been reported to affect cell apoptosis or the synthesis of the thyroid gland (Wu D.M. et al., 2018; Fan et al., 2019). It may be the case therefore that AS events affect the synthesis of the thyroid gland and, subsequently, the onset of puberty.

We also analyzed AS events in 36 genes that are known to be involved in puberty (Mechaly et al., 2009; Balasubramanian et al., 2010; Lomniczi et al., 2013; Plant, 2015b; Abreu and Kaiser, 2016; Chakradhar, 2018; Tang et al., 2018; Lents, 2019). Furthermore, the differential splicing of TAC1 can make it produce two isoforms (Page, 2005). The tachykinins encoded by these two isoforms have been shown to regulate the release of prolactin (PRL) *in vivo*, and these PRL secretagogues act on primary pituitary cells (Dupre et al., 2010). In the present study, we found that Cassette of *TAC1* was more specifically identified in pituitary in in-pubertal stage, compared with that of hypothalamus (Supplementary Table 15). The AltStart in *PGR* gene, which has been shown to be affected by pituitary-specific *Esr1* knockout (Sanchez-Criado et al., 2012), more exclusively occurred in pre-pubertal pituitary. The AltEnd of *FSHR*, which has been demonstrated to be involved in folliculogenesis (Candelaria et al., 2020), has been more specifically observed in ovaries in in-puberty, compared with hypothalamus. These finding and observations indicate that the ASs are expressed in a tissue-specific pattern based on their own functions during the onset of puberty. Moreover, each tissue is composed of different cells, and it is supposed that these ASs are cell specific in the cells of hypothalamus, pituitary, and ovary during the timing of puberty. A previous study has showed that cell-specific AS was essential for the function of cells (Lah et al., 2014; Gracida et al., 2016). Therefore, the more comprehensive profiles of AS changes and dynamics during the onset of puberty is supposed and proposed with the transcriptome data of single cells in HPO tissues.

In addition, the estrogen-related receptor alpha (*ESRRA*) gene encodes an orphan nuclear receptor that is involved in the release of GnRH in hypothalamus of marmoset monkey (Wahab et al., 2019). In the present study, the AltEnd event in *ESRRA* more exclusively occurred in pre-puberty, compared with post-puberty in ovary. It has been reported that *TACR3* directly regulates the release of GnRH and the onset of puberty (Hietamaki et al., 2017). In the present study, we found that Cassette of *TAC3R* was more specifically identified in post-puberty in pituitary, compared with that of pre-puberty. The AltEnd and AltStart occurring in *ESR1* gene, which is essential in response to normal onset of puberty and estrogen feedback (Cheong et al., 2015), more exclusively occurred in pre-puberty, compared with post-puberty in the ovary. These results suggest that ASs of genes exhibit in a specific pubertal stage during the timing of puberty in pigs.

Besides, we found that MXE and AltStart events highly occurred in HPO tissues during the pubertal transition of pigs. For example, *COL3A1*, which encodes the pro-α 1 chain of collagen III and is involved in the development of follicles (Yao et al., 2019), was identified as one of the genes that exclusively underwent MXE events in post-puberty. The *CYP19A1* gene, which is involved in the estradiol biosynthesis (Sutherland et al., 2017), uniquely harbors AltStart in hypothalamus. Alternatively, a previous study shows that *GH1* is involved in regulating onset of puberty (Hu et al., 2019), and we found that A5SS and MXE occur at different sites of *GH1* by interacting with four SFs at the post-puberty in the present study. *ACTB* has been demonstrated to regulate the reproductive function of the ovary (Hassanpour et al., 2019), and the *ACTB* gene specially shows AltEnd in ovary by intersecting with five SFs at the post-puberty (Supplementary Tables 16–18). These results suggest that SFs might be involved in the onset of puberty by regulating specific splicing of genes. These results provide AS level data for a clearer understanding of the mechanisms underlying onset of puberty in the future.

## Conclusion

Collectively, in this study, the AS dynamics and DEAS events were comprehensively profiled in hypothalamus, pituitary, and ovary across the pre-, in-, and post-pubertal stages in pigs. The related genes of DEAS were enriched in GnRH signaling pathway, thyroid hormone signaling pathway, oocyte meiosis, and oxytocin signaling pathway, which are all involved in the regulating and timing of puberty. Moreover, the ASs of *TAC1*, *TACR3*, *CYP19A1*, *ESR1*, *ESRRA*, and *FSHR* were likely to regulate the functions of certain HPO tissues during the onset of puberty. These findings may provide a step forward in our understanding of how molecular events, such as AS, can regulate the timing of puberty in mammals.

## Data Availability Statement

The datasets presented in this study can be found in online repositories. The names of the repository/repositories and accession number(s) can be found below: https://www.ebi.ac.uk/ena, PRJEB39754.

## Ethics Statement

The animal study was reviewed and approved by the Animal Care and Use Committee of the South China Agricultural University, Guangzhou, China (permit number: SCAU#2013-10). Written informed consent was obtained from the owners for the participation of their animals in this study.

## Author Contributions

XP, QL, YC, and XY conceived and designed this research. XP, QL, and DC acquired the biological samples and analyzed the data. WG, NL, YJ, and HZ originally derived the data and helped in the analysis. XP and QL drafted the manuscript. XY and YC critically revised the first draft. All authors reviewed and approved the final manuscript.

## Conflict of Interest

The authors declare that the research was conducted in the absence of any commercial or financial relationships that could be construed as a potential conflict of interest.

## Footnotes

1 https://sourceforge.net/projects/cash-program/

2 http://www.tbtools.com/

3 http://www.caspur.it/SpliceAidF/

## Supplementary Material

Supplementary Figure 1 The ovarian histology structure. **(A)** Pre-pubertal ovary. **(B)** In-pubertal ovary. **(C)** Post-pubertal ovary.

Supplementary Figure 2 Representative model of eight different alternative splicing types. The black solid lines represent connections between exons that have undergone alternative splicing. The colored dotted line refers to the original connection.

Supplementary Figure 3 Boxplots of number of AS and differentially expressed alternative splicing (DEAS). **(A)** AS in all pubertal tissue. **(B)** DEAS in hypothalamus. **(C)** DEAS in pituitary. **(D)** DEAS in ovary. **(E)** DEAS in pre-puberty. **(F)** DEAS in post-puberty. ^∗∗^FDR < 0.01, ^∗∗∗^FDR < 0.001.

Supplementary Figure 4 Specific alternative splicing (AS) events in the pubertal hypothalamus–pituitary–ovary (HPO) axis. **(A)** Stage-specific AS. **(B)** Tissue-specific AS.

## Abbreviations

AS: alternative splicing
DSG: differential splicing gene
DEAS: differentially expressed alternative splicing
A3SS: alternative 3′ splice site
A5SS: alternative 5′ splice site
MXE: mutually exclusive exons
SE (Cassette): skipping exons
Cassette multi: multi-cassette exons
IR: intron retention
AltStart: alternative start exon
AltEnd: alternative end exon
HPO: hypothalamus–pituitary–ovary axis.
