# Supplementary figures and images for: Alternative Splicing Dynamics of the Hypothalamus–Pituitary–Ovary Axis During Pubertal Transition in Gilts
Source: Front Genet. 2021 Apr 30;12:592669. doi: 10.3389/fgene.2021.592669 (PMC8120244; doi:10.3389/fgene.2021.592669)

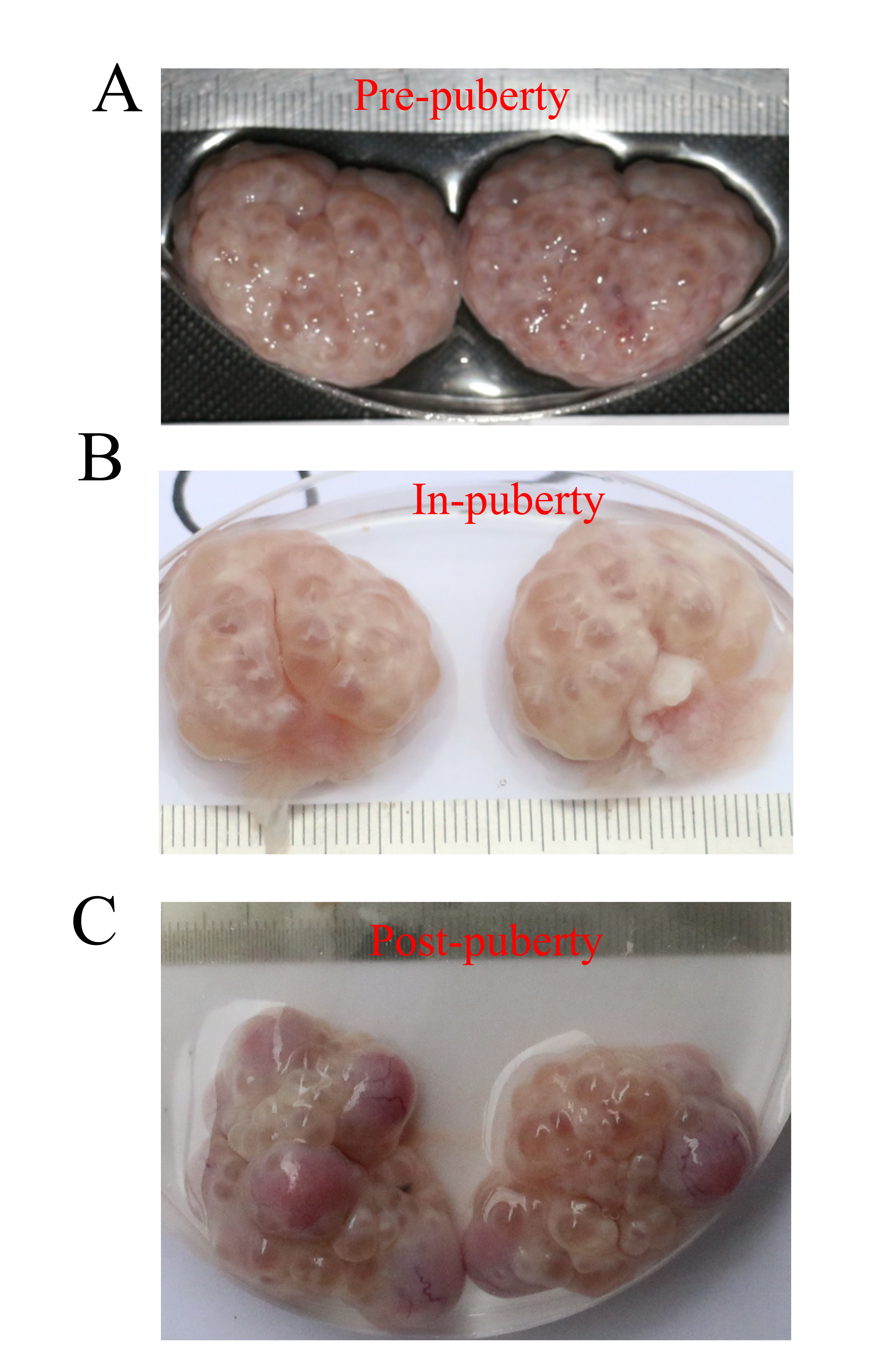

Supplement: Supplementary Figure 1 — The ovarian histology structure. (A) Pre-pubertal ovary. (B) In-pubertal ovary. (C) Post-pubertal ovary. [file Image_1.TIF]

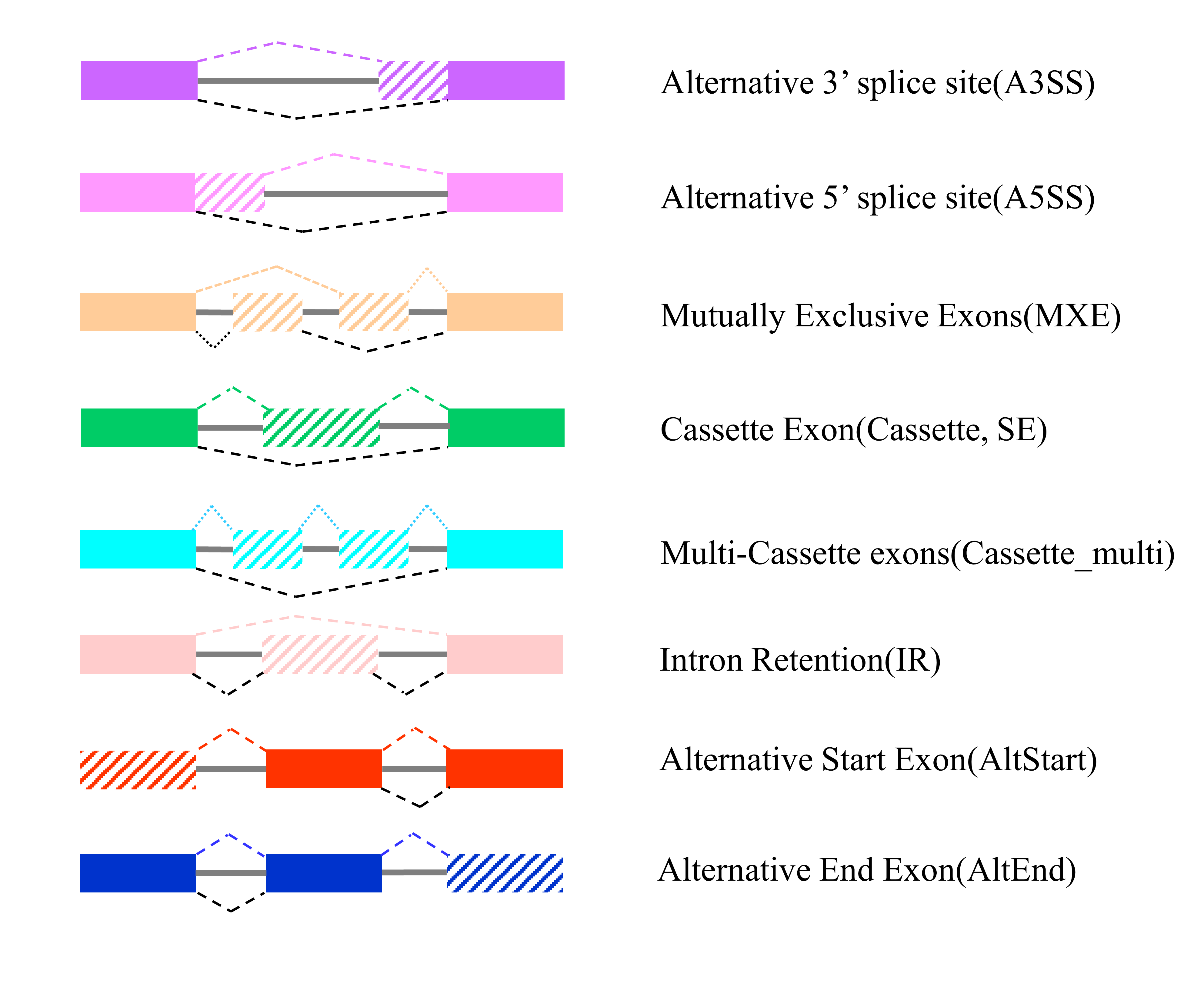

Supplement: Supplementary Figure 2 — Representative model of eight different alternative splicing types. The black solid lines represent connections between exons that have undergone alternative splicing. The colored dotted line refers to the original connection. [file Image_2.TIF]

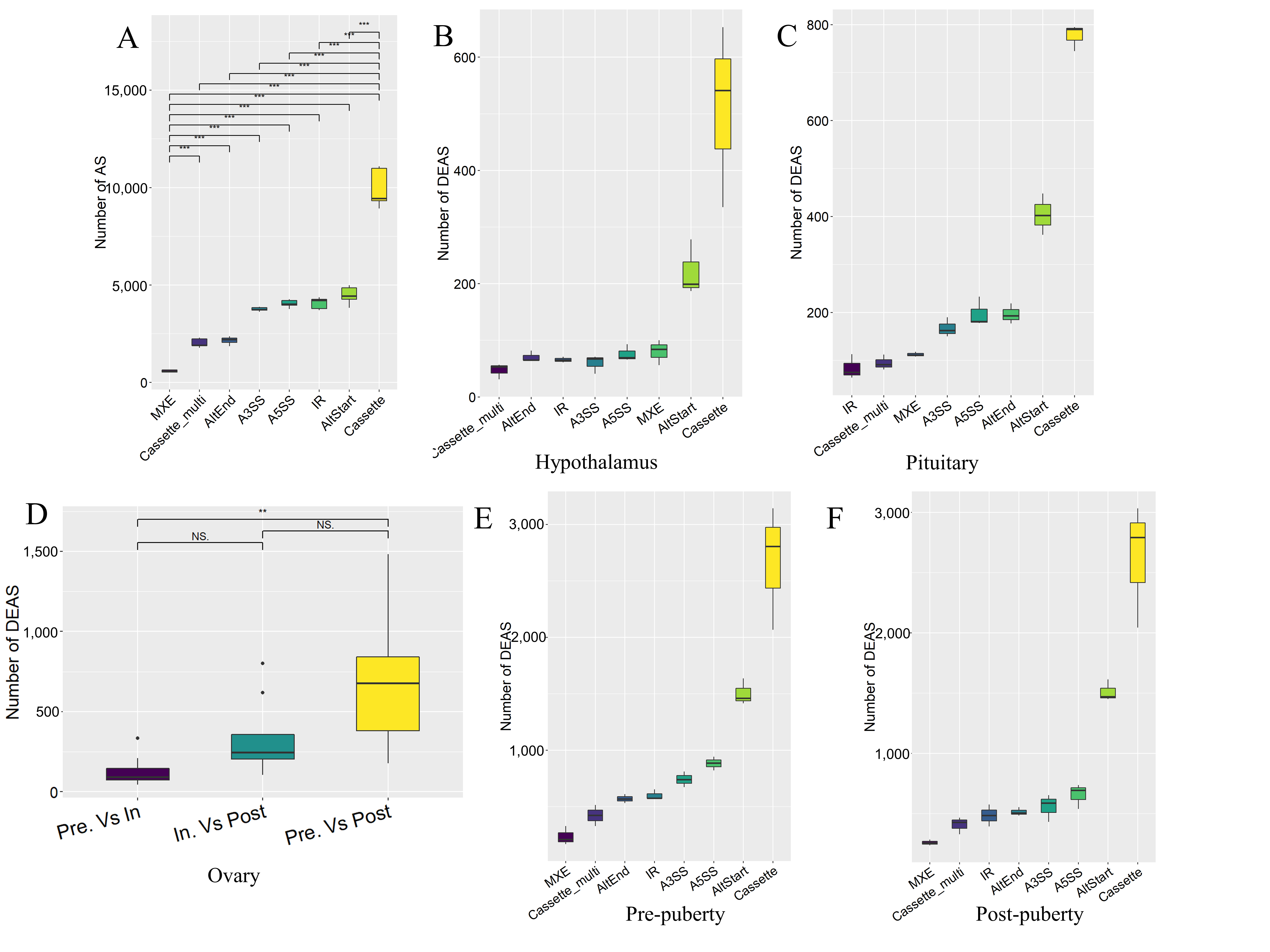

Supplement: Supplementary Figure 3 — Boxplots of number of AS and differentially expressed alternative splicing (DEAS). (A) AS in all pubertal tissue. (B) DEAS in hypothalamus. (C) DEAS in pituitary. (D) DEAS in ovary. (E) DEAS in pre-puberty. (F) DEAS in post-puberty. ∗∗FDR < 0.01, ∗∗∗FDR < 0.001. [file Image_3.TIF]

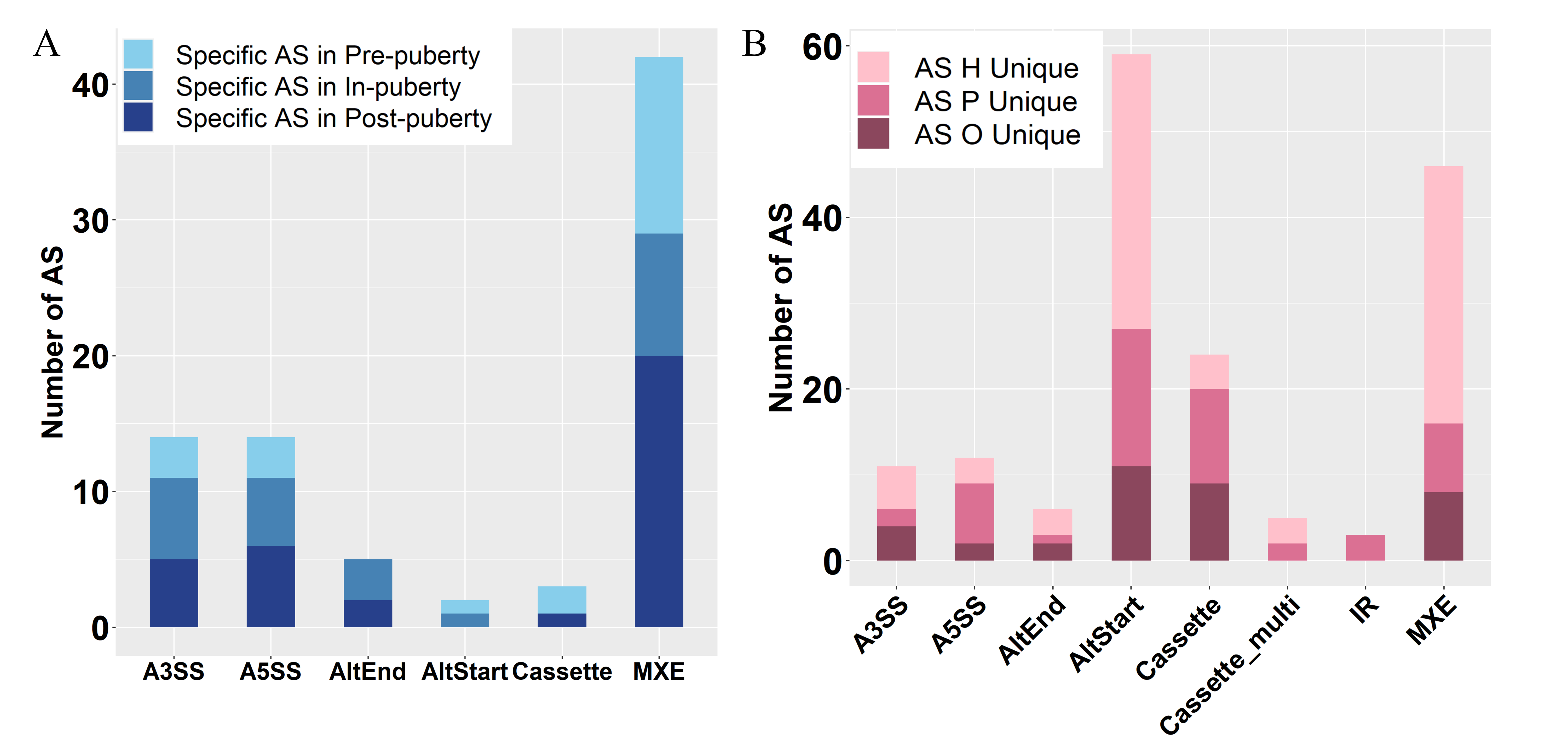

Supplement: Supplementary Figure 4 — Specific alternative splicing (AS) events in the pubertal hypothalamus–pituitary–ovary (HPO) axis. (A) Stage-specific AS. (B) Tissue-specific AS. [file Image_4.TIF]
